# How we manage medication-related osteonecrosis of the jaw

**DOI:** 10.1186/s40001-024-01912-6

**Published:** 2024-08-02

**Authors:** H. Byrne, S. O’Reilly, C. S. Weadick, P. Brady, R. Ni Ríordáin

**Affiliations:** 1 Cork University Dental School and Hospital, University College Cork, Cork, Ireland; 2 Cancer Research @UCC, College of Medicine and Health, University College Cork, Cork, Ireland; 3 Department of Medical Oncology, Cork University Hospital, Wilton, Cork, Ireland

**Keywords:** Medication-related osteonecrosis of the jaw, Dental oncology, Bone-modifying agents, Multidisciplinary patient care

## Abstract

Bone-modifying agents (BMAs) are integral to managing patients with advanced cancer. They improve quality of survival by reducing skeletal-related events, treating hypercalcaemia and chemotherapy-induced bone loss (Coleman in Clin Cancer Res 12: 6243s–6249s, 2006), (Coleman in Ann Oncol 31: 1650–1663, 2020). Two decades ago, medication-related osteonecrosis of the jaw (MRONJ) was first reported following BMA therapy (Marx in J Oral Maxillofac Surg 61: 1115–1117, 2003). The risk of MRONJ extends over a decade following BMA treatment with bisphosphonates, complicating dental care such as extractions. In addition, MRONJ has been reported following additional therapies such as antiangiogenic agents, cytotoxic agents, immunotherapy, and targeted agents. The use of BMAs in the curative and adjuvant cancer setting is increasing, consequently the implication of MRONJ is growing. Over the past 20 years, the literature has consolidated major risk factors for MRONJ, the pathophysiology and management strategies for MRONJ. Our review aims to document the development of MRONJ preventative and management strategies in cancer patients receiving a BMA. The authors advocate the incorporation of dental oncology strategies into contemporary cancer care, to optimise long-term quality of survival after cancer treatment.

## Introduction

Two decades ago, Marx described medication-related osteonecrosis of the jaw (MRONJ) as an emerging clinical entity in patients receiving bisphosphonate therapy [3, 4]. MRONJ is now an established condition defined by exposed bone that can be probed through an intra- or extra-oral fistula(e) in the maxillofacial region which has persisted for more than 8 weeks. The patient must have no history of radiation therapy or metastatic disease to the jaws and has had a current or previous treatment regime with antiresorptive therapy or antiangiogenic medications [4]. At present, bone-modifying agents (BMAs) are commonly prescribed to patients with prostate (85%), breast (70%), lung (40%), kidney (40%) cancer, and multiple myeloma (95%), to reduce the risk of skeletal-related events (SREs), chemotherapy-induced bone loss and to treat hypercalcaemia. Their effectiveness in reducing SREs has led to their integration into guideline based cancer care [1, 2, 5, 6]. Practising oncologists need to know how to identify at risk patients, and to integrate onco-dental strategies into multidisciplinary cancer care.

Over the past two decades, more than 1,500 articles have been published on MRONJ, reflecting its significant relevance. However, this increasing clinical relevance is compounded by the historical under-appreciation of dental diseases worldwide. In May 2021, the World Health Assembly (WHA) introduced the resolution WHA 74.5 as a global strategy to combat oral diseases. Dental disease remains the highest non-communicable disease over the life span of MRONJ [7, 8].

The purpose of this review is to provide practical guidance for oncologists prescribing BMAs, and to emphasise the need for further integration of dental oncology into cancer care.

### Clinical features of MRONJ

**Fig. 1:**
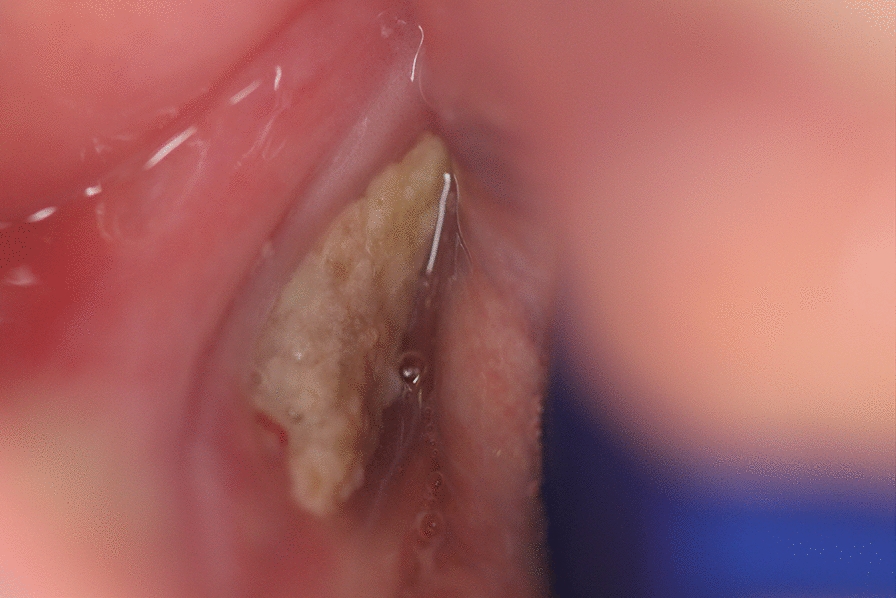
Is a clinical photograph of stage 2 (AAOMS) MRONJ—infected exposed mandible

**Fig. 2:**
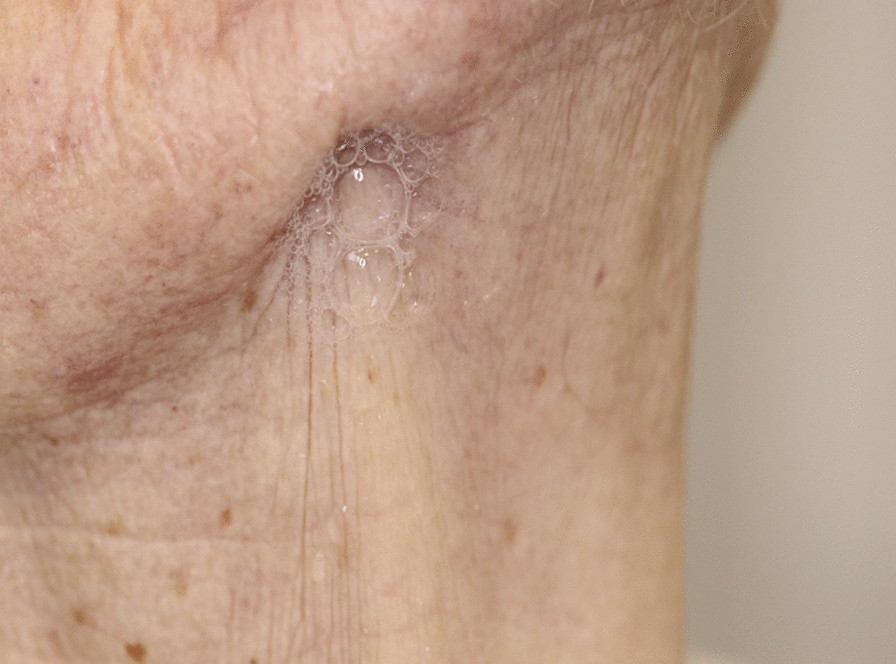
Is a clinical photo which highlights saliva extruding through the orocutaneous fistula in the right mandible (AAOMS Stage 3)

MRONJ can present with a variety of  clinical manifestations: exposed bone in the oral cavity, diffuse jaw pain, swelling, purulence, mobile teeth, fistula (intra- or extra-oral), sinusitis, pathological fracture, sensory changes, and maxillofacial deformity. The mandible is more commonly affected than the maxilla [9], (Fig. 1, 2). Radiographic analyses can aid with accurate size definition  [10]. In advanced cases, the cyclical nature of superinfection and remission is prominent along with pain and debilitation [11]. Mucosal breakdown is not always necessary to define MRONJ, and non-exposed regions of bone with exclusion of other disease processes can encompass the earliest stage of MRONJ [4].

MRONJ is staged most commonly using The American Association of Oral and Maxillofacial Surgery (AAOMS) staging systems, as shown in Table 1 [4]. McMahon et al. [12], Bedogni et al. [13] and Yoneda et al. [14] all require radiographic findings to calibrate staging.

**Table 1:** America Association of Oral and Maxillofacial (AAOMS) staging system for MRONJ [1]

Stage	Symptom	Clinical descriptor	Radiographic
Stage 0	Non-specific pain associated with teeth, mandible/maxilla or sinus Neurosensory dysfunction	No exposed bone Loose teeth excluding periodontal disease Intra- or extra-oral swelling	Non-specific alveolar bone loss Osteosclerosis Thickening of lamina dura
Stage 1	Asymptomatic	Exposed bone or probable through a fistula No inflammation or infection	Localised bone changes to the alveolus (bone loss, osteosclerosis)
Stage 2	Symptomatic	Exposed bone or probable through a fistula Evidence of inflammation or infection	Localised bone changes to the alveolus (bone loss, osteosclerosis)
Stage 3	Symptomatic	Exposed bone or probable through a fistula, beyond the alveolar bone Evidence of infection Extraoral fistula, oro-antral/oro-nasal communication, osteolysis to inferior mandibular border or sinus floor	Localised bone changes beyond the alveolus to the inferior mandibular border/sinus floor, pathological fracture
Patients at risk—bone-modifying agent administration	Asymptomatic	No exposed bone

**Fig. 3:**
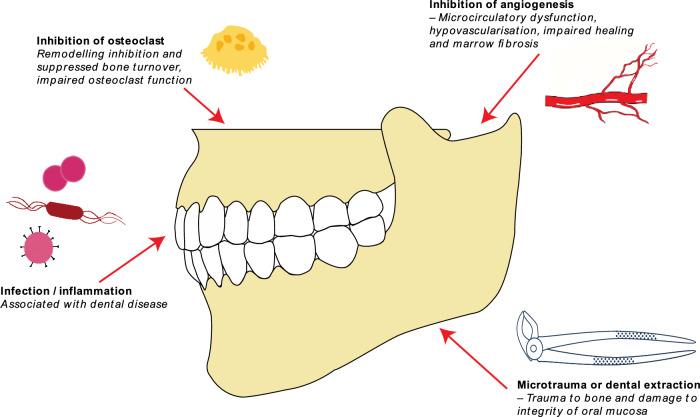
Highlights local factors implicated in the pathophysiology of MRONJ [4, 46]

**Fig. 4:**
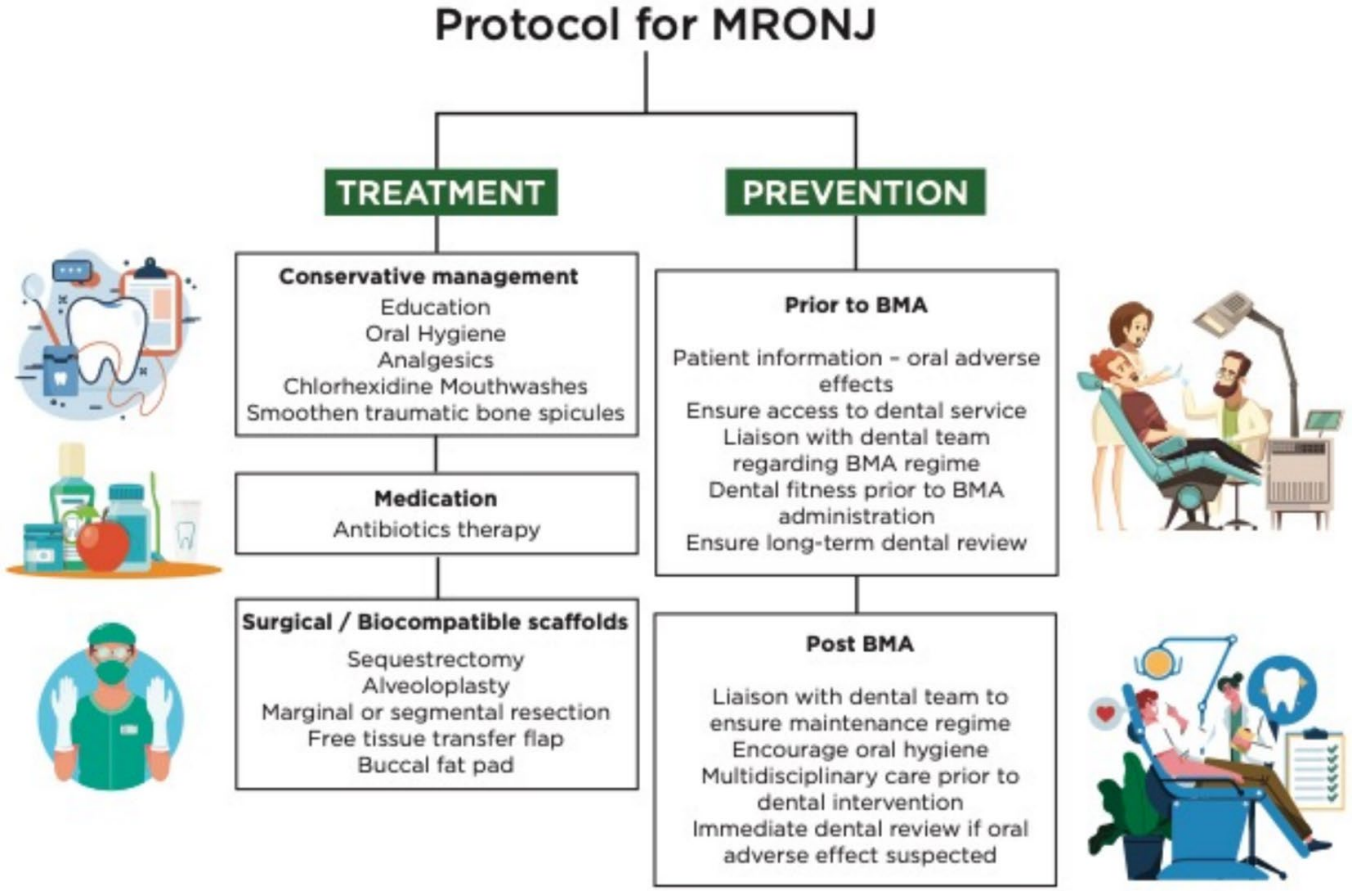
Depicts the management protocol for MRONJ and preventative strategies for patients intended for BMA prescription [4, 46]

**Fig. 5:**
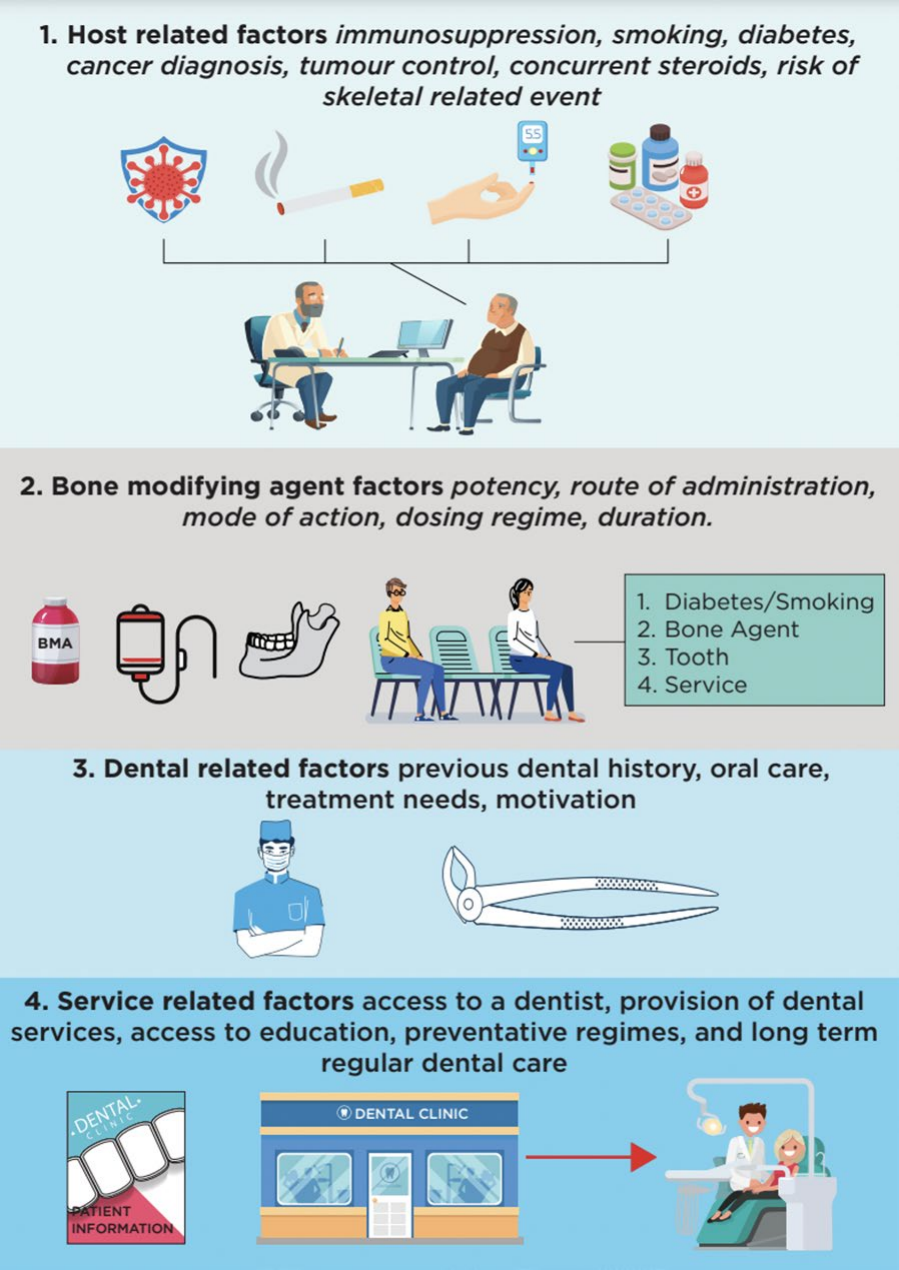
Depicts the factors affecting the patient’s journey and dental experience following the use of BMAs

Extension of MRONJ into the mandible or maxilla can result in large, exposed necrotic regions of bone with associated sensory deficit, pathological fracture, or an oroantral fistula in advanced stages (Fig. 2). MRONJ can affect a patient’s quality of life (QoL), including their functional capacity and psychological well-being [15]. The impact of MRONJ on QoL is poorly documented in the literature. Consequently, psychological impact, functional limitation, physical disability, and deformity are incorporated into the Oral Health Impact Profile-14 (OHIP-14) for the assessment of MRONJ on QoL. Psychological discomfort was rated as the greatest negative impact factor on the OHIP-14 questionnaire for MRONJ [15].

### Epidemiology

The prevalence of MRONJ in patients with cancer ranges from 0 to 12% [16] while the incidence rates in patients with metastatic bone disease on zoledronic acid therapy also vary from 1.5 to 6% [4]. Receptor activator of nuclear factor kappa-beta ligand (RANK-L) inhibitors have been used as alternatives to bisphosphonates, with a reported incidence of MRONJ following subcutaneous denosumab ranging between 0.7 and 6.7% [17–19]. Incorporation of BMAs into adjuvant therapy poses a long-term MRONJ risk for patients treated with a curative intent. MRONJ was prevalent between 0.00 and 1.8% in clinical trials when antiresorptives were used in the adjuvant setting. The long-term risk of MRONJ is also relevant to this cohort and provides a cautionary message for clinicians regarding dental risk in this group [20–26].

A meta-analysis by Srivastava et al. concluded that a higher incidence of MRONJ is associated with dual therapy rather than a single antiresorptive or antiangiogenic therapy. Examples of dual therapy include the combination of two BMAs, concurrent use of BMAs with steroid administration or cyclin-dependent kinase 4/6 inhibitors (CDK 4/6,) [4, 27]. The use of polypharmacy and variable regimes of antiresorptives, antiangiogenics, folate antagonist, targeted agents and immunotherapies for cancer, implies a globalised difficulty to refine the exact risk of MRONJ [28–35].

### Pathophysiology

The pathophysiology of MRONJ remains unproven however significant advances have been made over the past two decades to refine vital pathological processes [36], (Fig. 3). Alveolar bone is a highly active bone that is capable of rapid remodelling. The predilection of the jaw reflects the proximity of the bone to the oral mucosa and the oral microbiome. Dental infection and inflammatory processes have been implicated in MRONJ [37]. Pre-existing dental infection plays a significant role, which predisposes patients to MRONJ, which remains independent to a tooth extraction [38].

Site-specific jaw involvement has been attributed to bone remodelling inhibition, suppressed bone turnover, inflammation, infection, direct cellular toxic effects, angiogenesis inhibition, innate or acquired immune dysfunction, and genetic predisposition. The typical histological picture of osteonecrosis includes marrow fibrosis, hypovascularisation, and bacterial colonisation with inflammation [36, 39, 40]. Severe dental disease and infection coupled with osteoclast inhibition are predominant initiators of MRONJ [37, 41–45]. It is likely that its pathogenesis is multifactorial in a susceptible host exposed to BMA therapy.

Systemic risk factors contribute to the development of MRONJ in a susceptible patient. This immune compromise is compounded by acquired immune dysfunction seen in patients with medical comorbidities such as diabetes mellitus, rheumatoid arthritis, or immunocompromised states such as corticosteroid use and cytotoxic chemotherapy [47–49].

### Treatment of MRONJ

There is no definitive cure for MRONJ; however, the ultimate goal is mucosal closure to optimise quality of life.

The management of MRONJ begins with a thorough medical, social, and dental history. Specific BMA regimens and exacerbating factors must be confirmed. Initial conservative treatment strategies play a vital role in the management of all stages of MRONJ and are a valuable adjunct to both medical and surgical treatments [50, 51]. Ruggiero et al. 2014 devised a treatment strategy based on staging and symptoms [16]. Antiseptic mouth wash, oral hygiene, patient education, and regular dental review (3-monthly) are basic conservative regimes for all stages of MRONJ [4, 41]. Stage 0 or 1 requires symptomatic control of pain and meticulous oral hygiene measures. Debridement of sharp spicules of necrotic bone may be also warranted [4], (Fig. 4).

### Medical intervention

The medical management of MRONJ remains a viable option for patients. Data from a double-blinded, randomised control trial on the efficacy of teriparatide for the treatment of MRONJ reported higher MRONJ resolution rates in the treatment arm compared the placebo at 52 weeks [52]. Sim et al. noted a 45.4% resolution of MRONJ (radiographic and clinical improvement) compared to 33% resolution in the placebo group over 12 months using teriparatide [52], demonstrating the short-term use of teriparatide could be both an efficacious and safe medical strategies for the management of MRONJ [52]. The side effects profile of teriparatide includes nausea, anorexia, renal dysfunction and musculoskeletal pain which can be limiting factors [52]. The incidence of osteosarcoma increased in rats following the administration of teriparatide, however this dose-dependency was observed in toxicology studies in rats only [53]. Low dosing regimens prior to and following surgical interventions can offer superior outcomes to surgical intervention alone [54, 55]. A pathobiological explanation for the effects of teriparatide on MRONJ is derived from its osteoanabolic properties stimulating osteoblasts, reducing exposed bone volume and increasing mucosal coverage [52, 56]. Pentoxifylline and tocopherol (PENTE) has been originally cited for their benefits in the management of osteoradionecrosis [57]. Data were limited to 3 observational studies and 2 abstracts for the use of pentoxifylline and tocopherol in the medical management of MRONJ, however promising results were yielded from all studies [58]. These studies showed that PENTE reduced painful symptoms and promoted new bone formation in a well-tolerated regime of PENTE for the management of MRONJ [39]. Patel et al. concluded the prophylactic use of PENTE prior to a dental extraction was superior to placebo when used in patients at risk of osteoradionecrosis [40]. The role of antibiotics relates directly to prominent micro-organisms that reside in the biofilm in the oral cavity, Actinomyces species [29, 50]. Beta lactams remain the antibiotic of choice [59]. Hospitalisation and intravenous antibiotics may be required in indicated clinical and systemic condition.

Bio-stimulation modalities such as hyperbaric oxygen, low-intensity laser, and medical ozone therapy, offer no definitive evidence to support cure or cessation of progression of MORNJ [40, 47, 49, 60–63]. Recombinant human bone morphogenic protein-2 is a transforming growth factor beta and approved as a bone graft substitute. Its use has been suggested in the treatment of MRONJ, however further research is implicated to define its efficacy in this cohort [64, 65]. Photobiomodulation (PBM) therapy has evoked interest in the treatment of acute oral mucositis following radiation therapy in head and neck oncology patients. The WALT position paper 2022 also reviewed its application in the treatment of MRONJ [66]. PBM has been incorporated into MRONJ treatment regimens, proving beneficial as an adjunct to surgical and antibiotic therapy [67]. The use of low-level laser therapy was proposed following the WALT report for the management of MRONJ [66]. Inflammatory suppression signalling and low-level laser therapy have shown promising results in in vitro studies for gingival wound healing and subsequent bone regeneration following tooth extraction in zoledronate treated specimens [68]. Sole medical management strategies for MRONJ have been based on case reports and small clinical trials, to optimise the potential synergistic effects of multiple medical management strategies for MRONJ particularly in the maxilla where complex surgical intervention was contraindicated in oncology patients [69]. A multimodality care algorithm was proposed including chlorhexidine with exposed bone, antibiotics when clinically required, PBM delivered by medical and patient at home LED-based PBM device, teriparatide (20 µg per day for 2 months), and pentoxifylline and tocopherol [69].

### Surgical intervention

Surgical management was initially used to palliate MRONJ, which was unresponsive to conservative and medical management [46]. Surgical management is introduced earlier in the treatment cycle in less aggressive surgical interventions such as superficial debridement, sequestrectomy, and alveoloplasty to achieve necrotic bone removal and primary mucosal closure. Tensionless mucosal flaps are imperative to optimise healing, help stimulate angiogenesis and basal lamina signalling pathways [4, 9, 70–73]. CT-based surgical planning plays a vital role prior to surgical management which helps to accurately define the affected, bone defect [74]. Marcianò et al., reported on a 10-year retrospective, single-centre study on surgical outcomes for MRONJ [75]. A surgical decision tree was devised based on their findings of 128 MRONJ surgeries, in both curative and palliative cases, to guide the hard and soft tissue management of MRONJ. They advocated the use of piezoelectric devices in saucerisation and additional osteoplasty measures in more aggressive alveolar-block resections to eliminate residual bone asperity [75]. Radical decortication, submarginal bone resection, rim mandibulectomy, and local resection are used for progressively advanced stage surgical procedures for MRONJ [76]. Replacement of tissue with biocompatible scaffolds and osteogenic potentiator cells such as buccal fat pad, and restoration of form and function using free tissue transfer flaps may be utilised following resection [61]. Free tissue transfer flaps have been used to address large defects in stage 3 patients and case studies have reported promising success rates of 96% [76]. Fibula free flaps have been favoured in these oncology patients due to low incidence of primary bone malignancy or metastatic bone disease [77]. Soft tissue management in deficient cases may require advancement of mucoperiosteal flaps, mylohyoid flaps or pedicled buccal fat pad flaps to ensure primary closure and provide additional protection against wound dehiscence. The use of locally derived growth factors such as Platelet Rich in Growth Factors (PRGF) and Platelet-Rich Plasma (PRP) remains controversial [9]. Surgical intervention can be associated with a deterioration in a patient’s clinical status which must also be appreciated by clinicians [78]. Case selection and identification of exacerbation factors are important pre-surgical considerations [79].

The literature offers a heterogenous range of treatment regimes, which often lack standardisation, leading to uncertainty about effective treatment modalities. Migliorati et al. reported on an array of treatment modalities in oncology patients on a BMA. Medical therapy resulted in resolution of MRONJ in 17.6% (*n* = 120), surgical debridement in 17.3% (*n* = 118) and surgical flap and / or resection in 46.3% (*n* = 316). Mucosal healing was reported in 56.2% (*n* = 380) [80]. Host, medical, dental and treatment variabilities contribute to the heterogenous response to therapies and management challenges [80, 81] (Fig. 5).

### Prevention of MRONJ

#### Primary prevention of MRONJ—before and during treatment with a bone-modifying agent

Preventative dental therapy prior to BMA has been shown to reduce the incidence of MRONJ compared to oncology populations without a pre-therapeutic preventative dental programme [51, 82–84]. Most recent documents to guide MRONJ preventative regimes include the Cochrane Oral Health, 2022 (3rd version) [85], American Association of Oral and Maxillofacial Surgeons, 2022 (4th version) [4] and Italian Society of Maxillofacial Surgery / Italian Society of Oral Pathology and Medicine, 2023 (3rd version) [86] which define timelines for dental review regimes (3-monthly) for patients on a BMA [85], MRONJ risk factor identification [4], the use of antibiotic therapy and primary closure following an extraction [86, 87]. The placement of implants in high-risk oncology patients following BMA therapy was contra-indicated [4, 86]. Another cornerstone document which provides guidance for the prevention and management of MRONJ is the Multinational Association for Supportive Care in Cancer/International Society of Oral Oncology/American Society of Clinical Oncology (MASCC/ISOO/ASCO) Clinical Practical Guidance, 2019 (1st version) [88]. This document was produced by an expert panel of oncologist based on 10 randomised control trials. It reinforces the necessity of multidisciplinary co-ordination of care between specialities, does not advocate the use of drug holidays, guides MRONJ risk stratification and could not conclude on the role of prophylactic antibiotic therapy with dental extractions [88, 89].

Primary prevention predominantly aims to reduce or eliminate dental risk factors [90]. Bramati et al. demonstrated a decreased incidence of MRONJ following a dental preventative programme, from 11 to 7% [91]. Timely dental care screenings facilitate the removal of non-restorable teeth or those with periapical infection, address periodontal disease, commencement of a stringent hygiene protocol to maintain a high standard of oral health, occlusal adjustment, parafunctional protection, atraumatic-fitting dental prostheses, topical remineralising therapy and caries control [46, 51, 82, 83]. MRONJ risk reduction, smoking cessation, dental hygiene education, and promotion of 3-monthly dental reviews must be addressed in the primary preventative stage [85]. It is important to note that for a new adult oncology patient, co-ordination of dentistry predominantly depends on patient-driven attendance and self-reliance [92]. The importance of routine dental assessments at the beginning of systemic anti-cancer therapy involving BMAs should be integrated into routine cancer care [93]. The role of the dental auxiliaries, such as dental hygienists, in regular professional supragingival and subgingival scaling has proven to be a beneficial preventative strategy [51]. Early detection of dental diseases throughout the course of BMA treatment can reduce the requirement for invasive dental procedures [90]. A randomised control trial on the impact of preventative dental treatment strategies and 3-monthly dental reviews following BMA administration were associated with a 2.59 reduced risk reduction for MRONJ [94].

#### Secondary prevention of MRONJ

Secondary prevention aims to facilitate the early detection of MRONJ. Regular routine dental care permits the detection of MRONJ in earlier stages of development, which tend to have more predictable outcomes and reduced morbidity [90]. Coordination of regimented dental surveillance before, during and after patient exposure to a BMA minimises the risk of MRONJ [83, 85]. Delaying the BMA may be implemented if systemic factors permit to achieve dental fitness [95].

Drug holidays have yet to be proven as definitively effective strategies to prevent MRONJ; their benefits are unclear, and the literature remains divided [4, 90]. The term drug holiday can pose misconceptions related to a temporary suspension of BMA prior to a dental intervention or the cessation of BMA due to an MRONJ diagnosis [90]. The former is only accepted as a temporary suspension, and the length of a drug holiday is specific to each medication depending on mode of action and half-life [97]. The foundation of a drug holiday is despite the 11.2 year half-life of oral bisphosphonates, bone marrow stem cells and osteoclast precursors have the ability to regenerate to sufficient numbers to remodel and renew bone during the healing process. However, an effective drug holiday is not achievable in cancer patients for intravenous bisphosphonates or subcutaneous denosumab (120 mg/3 monthly) for skeletal-related events because of the more rapid and gradual bone depletion of bone marrow osteoclast precursor cells and potency of the therapy in the oncology setting [46]. Drug holidays for osteoporotic patients should be utilised under the prescribing physician’s responsibility; however, this preventative strategy may be ineffective for the oncology patient. A recent randomised clinical feasibility trial conducted with antiresorptive therapies demonstrated no prevention of MRONJ development within a 4-month drug holiday after surgical tooth removal in the oncology cohort. The authors noted a decline in patient-reported health outcomes during the drug holidays compared with drug continuation [98]. There are no randomised control trials in this field, and despite low-quality evidence, the benefit of a drug holiday for the prevention of MRONJ is not warranted [98–100]. There are no bone turnover biomarkers which are currently effective for detection or monitoring of MRONJ. C-terminal crosslinking telopeptide, vascular endothelial growth factor activity, endocrine function, and parathyroid hormone levels have yet to be proven as reliable indicators in this field [4].

Current BMA prescription trends in breast and prostate cancer with bone metastases, have advocated the de-escalation of antiresorptive therapy usually two years after cancer treatment. This step towards reducing the administration of antiresorptive drugs has been shown to be safe, and does not change the risk or incidence of SREs, health-related quality of life (HRQL)-physical subdomain, pain and symptomatic skeletal-related events (SSE)-free survival [101, 102]. Data appears to be more confounding for bisphosphonates compared to RANK-ligand inhibitors [101, 103].

### Team-work

It is important to address healthcare inadequacies such as the burden of dental disease in vulnerable cohorts, to create reciprocal interactions and improve healthcare services [104]. Overcoming challenges in healthcare is a universal dilemma that is overlooked across a vast array of disciplines [105–107].

### Future directions

In the absence of proactive dental oncology protocols, we anticipate that MRONJ will become increasingly relevant in the coming decades. To date, 1500 research articles have been published in the literature on MRONJ with no definitive consensus about the management of MORNJ, which heightens the importance of the role of preventative care and regular dental review for these patients. The level of evidence for management strategies is predominantly derived from cohort and case–control studies as well as systematic reviews of them. The restriction of lower-quality evidence in the sphere of MRONJ still defines the progression of information regarding its aetiology, epidemiology and management [85]. The authors appreciate the challenges of randomised control trials of MRONJ and valid ethical concerns.

An additional exacerbating factor in recent high-risk cohorts is the use of drugs that increase the existing toxicity of BMAs. For example, CDK 4/6 inhibitors commonly used to treat hormone sensitive breast cancers, can increase MRONJ risk when prescribed in conjunction with BMAs [27].

The oncology cohort remains the most vulnerable group for potential MRONJ development, both systemically and pharmacologically. The disease entity relies on preventative protocols before BMA therapy to reduce the rate of MRONJ development. Treatment protocols have remained relatively similar over the past 20 years as conservative first-line treatments to help control smaller regions of exposed bone. Surgical intervention plays a role in the management of MRONJ; however, this can be unpredictable and can be attributed to the multifactorial nature of the host and disease entity [4, 71, 73, 108]. Medical management can offer benefit to patients with MRONJ, in particular the use of teriparatide in the management of MRONJ [52, 56].

For practising oncologists today, MRONJ is a risk for patients in both palliative and curative settings. This review highlights the importance of dental preventive strategies and the array of current management strategies under investigation.

## Recommendations

### Take home guidance for the treating oncologist

Before treatment with a bone-modifying agent

- Patient information—risk of MRONJ [4]
- Liaison with dental team prior to BMA administration [51]
- Facilitate dental assessment [92, 93]
- Achieve dental fitness prior to BMA [82]
- Ensure dental review regime in place [90]

During treatment with a bone-modifying agent

- Liaison with dental team to ensure maintenance regime [51]
- Encourage denture hygiene, topical fluoride therapy, professional cleanings and interdental cleaning aids [51]
- Multidisciplinary care prior to dental intervention [90]
- Immediate dental review if oral adverse effect suspected [4]

After treatment with a bone-modifying agent

- Liaison with dental team to ensure maintenance regime (3-monthly) [90]
- Multidisciplinary care prior to dental intervention [4]
- Patient information and impact of BMA half-life on dental care [85]

Suspected MRONJ

- Recommendation of chlorhexidine mouthwash [16]
- Prompt referral to an oral surgeon or maxillofacial unit [51]
- Correspondence including antiresorptive/angiogenic history [9]

### Commonly asked questions in the oncology cohort

**Who should I refer to if a dental extraction is needed?**

Prior to a BMA, the patient’s general dental practitioner is suitable to extract a tooth.

After receiving a BMA, a dentist, oral surgeon (or maxillofacial surgeon) is the most appropriate [109].

**Do all patients need antibiotics?**

For patients with a previous or current history of BMAs and a requirement for a tooth extraction; pre- and post-operative antibiotics should be considered by the dental surgeon. Penicillin-based antibiotics (amoxicillin ± clavulanic acid) or clindamycin in patients with penicillin allergies are recommended [4, 85, 90, 96].

**Do drug holidays work?**

Uncertainty remains regarding the efficacy of a drug holiday; however, the literature favours its ineffective nature in the prevention of MRONJ in the oncology cohorts. The authors do not advocate a drug holiday in this specific cohort [4, 46, 90, 98–100]. Delaying BMA therapy if systemically permitted, may allow patients to reach dental fitness and reduce the risk of MRONJ [4].

**Is it ever safe to reintroduce BMAs in patients with a history of MRONJ?**

Case-based assessment is necessary in conjunction with communication with the treating dental surgeon to determine the status of MRONJ and the systemic requirements of the BMA. There are no data available to answer this clinical question. If avoidable, the reintroduction of the BMA would be ill-advised. The development of a real-world data base of such a cohort would be helpful.

## Data Availability

The data used to support findings and information in this article are included within the article.

## References

[1] Coleman RE. Clinical features of metastatic bone disease and risk of skeletal morbidity. Clin Cancer Res. 2006;12(20 Pt 2):6243s-s6249. 17062708 10.1158/1078-0432.CCR-06-0931

[2] Coleman R, Hadji P, Body JJ, Santini D, Chow E, Terpos E, et al. Bone health in cancer: ESMO Clinical Practice Guidelines†. Ann Oncol. 2020;31(12):1650–63. 32801018 10.1016/j.annonc.2020.07.019

[3] Marx RE. Pamidronate (Aredia) and zoledronate (Zometa) induced avascular necrosis of the jaws: a growing epidemic. J Oral Maxillofac Surg. 2003;61(9):1115–7. 12966493 10.1016/S0278-2391(03)00720-1

[4] Ruggiero SL, Dodson TB, Aghaloo T, Carlson ER, Ward BB, Kademani D. American association of oral and maxillofacial surgeons’ position paper on medication-related osteonecrosis of the Jaws-2022 update. J Oral Maxillofac Surg. 2022;80(5):920–43. 35300956 10.1016/j.joms.2022.02.008

[5] von Moos R, Costa L, Gonzalez-Suarez E, Terpos E, Niepel D, Body JJ. Management of bone health in solid tumours: from bisphosphonates to a monoclonal antibody. Cancer Treat Rev. 2019;76:57–67. 31136850 10.1016/j.ctrv.2019.05.003

[6] D’Oronzo S, Coleman R, Brown J, Silvestris F. Metastatic bone disease: pathogenesis and therapeutic options: Up-date on bone metastasis management. J Bone Oncol. 2019;1(15):100205. 10.1016/j.jbo.2018.10.004 PMC6429006 30937279

[7] World Health Assembly 66. Follow-up to the Political Declaration of the High-level Meeting of the General Assembly on the Prevention and Control of Non-communicable Diseases. Report No.: WHA66.10. 2013. https://apps.who.int/iris/handle/10665/150161. Accessed 12 Dec 2022.

[8] Agrasuta V, Thumbuntu T, Karawekpanyawong R, Panichkriangkrai W, Viriyathorn S, Reeponmaha T, et al. Progressive realisation of universal access to oral health services: what evidence is needed? BMJ Glob Health. 2021;6(7):e006556. 34257139 10.1136/bmjgh-2021-006556 PMC8278897

[9] Marcianò A, Rubino E, Peditto M, Mauceri R, Oteri G. Oral surgical management of bone and soft tissues in MRONJ treatment: a decisional tree. Life (Basel). 2020;10(7):99. 32610675 10.3390/life10070099 PMC7399969

[10] Moreno-Rabié C, Lapauw L, Gaêta-Araujo H, Ferreira-Leite A, Coucke W, van den Wyngaert T, et al. Radiographic predictors for MRONJ in oncologic patients undergoing tooth extraction. Sci Rep. 2022;4(12):11280. 10.1038/s41598-022-15254-y PMC9252989 35789184

[11] Filleul O, Crompot E, Saussez S. Bisphosphonate-induced osteonecrosis of the jaw: a review of 2,400 patient cases. J Cancer Res Clin Oncol. 2010;136(8):1117–24. 20508948 10.1007/s00432-010-0907-7 PMC11828172

[12] McMahon RE, Bouquot JE, Glueck CJ, Griep JA, Adams WR, Spolnik KJ, et al. Staging bisphosphonate-related osteonecrosis of the jaw should include early stages of disease. J Oral Maxillofac Surg. 2007;65(9):1899–900. 17719423 10.1016/j.joms.2007.04.021

[13] Bedogni A, Fusco V, Agrillo A, Campisi G. Learning from experience. Proposal of a refined definition and staging system for bisphosphonate-related osteonecrosis of the jaw (BRONJ). Oral Dis. 2012;18(6):621–3. 22353421 10.1111/j.1601-0825.2012.01903.x PMC3443365

[14] Japanese Allied Committee on Osteonecrosis of the Jaw, Yoneda T, Hagino H, Sugimoto T, Ohta H, Takahashi S, et al. Antiresorptive agent-related osteonecrosis of the jaw: Position Paper 2017 of the Japanese Allied Committee on Osteonecrosis of the Jaw. J Bone Miner Metab. 2017;35(1):6–19. 28035494 10.1007/s00774-016-0810-7

[15] Caminha RDG, Alcantara PL, Carvalho CG, Reia VCB, Capelozza ALA, da Santos PSS. The impact of medication-related osteonecrosis of the jaws on the quality of life in cancer patients. J Clin Exp Dent. 2020;12(8):e725-9. 32913568 10.4317/jced.56307 PMC7474943

[16] Ruggiero SL, Dodson TB, Fantasia J, Goodday R, Aghaloo T, Mehrotra B, et al. American Association of Oral and Maxillofacial Surgeons position paper on medication-related osteonecrosis of the jaw–2014 update. J Oral Maxillofac Surg. 2014;72(10):1938–56. 25234529 10.1016/j.joms.2014.04.031

[17] AlRowis R, Aldawood A, AlOtaibi M, Alnasser E, AlSaif I, Aljaber A, et al. Medication-Related Osteonecrosis of the Jaw (MRONJ): a review of pathophysiology, risk factors, preventive measures and treatment strategies. Saudi Dent J. 2022;34(3):202–10. 35935720 10.1016/j.sdentj.2022.01.003 PMC9346931

[18] Coleman R, Finkelstein DM, Barrios C, Martin M, Iwata H, Hegg R, et al. Adjuvant denosumab in early breast cancer (D-CARE): an international, multicentre, randomised, controlled, phase 3 trial. Lancet Oncol. 2020;21(1):60–72. 31806543 10.1016/S1470-2045(19)30687-4

[19] Ruggiero SL, Dodson TB, Assael LA, Landesberg R, Marx RE, Mehrotra B, et al. American Association of Oral and Maxillofacial Surgeons position paper on bisphosphonate-related osteonecrosis of the jaws–2009 update. J Oral Maxillofac Surg. 2009;67(5 Suppl):2–12. 19371809 10.1016/j.joms.2009.01.009

[20] Coleman RE, Collinson M, Gregory W, Marshall H, Bell R, Dodwell D, et al. Benefits and risks of adjuvant treatment with zoledronic acid in stage II/III breast cancer. 10 years follow-up of the AZURE randomized clinical trial (BIG 01/04). J Bone Oncol. 2018;13:123–35. 30591866 10.1016/j.jbo.2018.09.008 PMC6303395

[21] Gnant M, Mlineritsch B, Schippinger W, Luschin-Ebengreuth G, Pöstlberger S, Menzel C, et al. Endocrine therapy plus zoledronic acid in premenopausal breast cancer. N Engl J Med. 2009;360(7):679–91. 19213681 10.1056/NEJMoa0806285

[22] Kizub DA, Miao J, Schubert MM, Paterson AHG, Clemons M, Dees EC, et al. Risk factors for bisphosphonate-associated osteonecrosis of the jaw in the prospective randomized trial of adjuvant bisphosphonates for early-stage breast cancer (SWOG 0307). Support Care Cancer. 2021;29(5):2509–17. 32929540 10.1007/s00520-020-05748-8 PMC7956914

[23] Paterson AHG, Anderson SJ, Lembersky BC, Fehrenbacher L, Falkson CI, King KM, et al. Oral clodronate for adjuvant treatment of operable breast cancer (National Surgical Adjuvant Breast and Bowel Project protocol B-34): a multicentre, placebo-controlled, randomised trial. Lancet Oncol. 2012;13(7):734–42. 22704583 10.1016/S1470-2045(12)70226-7 PMC4970583

[24] Valachis A, Polyzos NP, Coleman RE, Gnant M, Eidtmann H, Brufsky AM, et al. Adjuvant therapy with zoledronic acid in patients with breast cancer: a systematic review and meta-analysis. Oncologist. 2013;18(4):353–61. 23404816 10.1634/theoncologist.2012-0261 PMC3639520

[25] Vidula N, Greenberg S, Petrillo L, Hwang J, Melisko M, Goga A, et al. Evaluation of disseminated tumor cells and circulating tumor cells in patients with breast cancer receiving adjuvant zoledronic acid. NPJ Breast Cancer. 2021;7(1):113. 34489453 10.1038/s41523-021-00323-8 PMC8421499

[26] von Minckwitz G, Möbus V, Schneeweiss A, Huober J, Thomssen C, Untch M, et al. German adjuvant intergroup node-positive study: a phase III trial to compare oral ibandronate versus observation in patients with high-risk early breast cancer. J Clin Oncol. 2013;31(28):3531–9. 23980081 10.1200/JCO.2012.47.2167

[27] Fusco V, Alessio M, Guglielmini PF, Vincenti M, Fasciolo A, Rossi M. Is Medication-Related Osteonecrosis of the Jaws (MRONJ) Associated to Cyclin-Dependent Kinase (CDK) 4/6 inhibitors? A Word of Cautiousness. Comment on Marcianò et al. medication-related osteonecrosis of the jaws and CDK4/6 Inhibitors: a recent association. Int J Environ Res Public Health. 2020; 17: 9509. Int J Environ Res Public Health. 2021; 18(19): 10143. 10.3390/ijerph181910143 PMC8508584 34639444

[28] Hallmer F, Bjarnadottir O, Götrick B, Malmström P, Andersson G. Incidence of and risk factors for medication-related osteonecrosis of the jaw in women with breast cancer with bone metastasis: a population-based study. Oral Surg Oral Med Oral Pathol Oral Radiol. 2020;130(3):252–7. 32536575 10.1016/j.oooo.2020.04.808

[29] Petrovic M, Jelovac DB, Antic S, Antunovic M, Lukic N, Sabani M, et al. Medication-related osteonecrosis of the jaws: two center retrospective cohort studies. Biomed Res Int. 2019;18(2019):8345309. 10.1155/2019/8345309 PMC6442486 31011580

[30] Jung S, Kim J, Park JH, Kim KY, Kim HJ, Park W. A 5-year retrospective cohort study of denosumab induced medication related osteonecrosis of the jaw in osteoporosis patients. Sci Rep. 2022;12(1):8641. 35606457 10.1038/s41598-022-11615-9 PMC9126865

[31] Milosavljević M, Jovanović M, Folić M, Živić M, Zdravković D, Veličković S, et al. Possible association of methotrexate use with osteonecrosis of the jaw: systematic review. J Stomatol Oral Maxillofac Surg. 2022;123(5):e458–63. 35306206 10.1016/j.jormas.2022.03.012

[32] Owosho AA, Liang STY, Sax AZ, Wu K, Yom SK, Huryn JM, et al. Medication-related osteonecrosis of the jaw (MRONJ): an update on the Memorial Sloan Kettering Cancer Center (MSKCC) experience and the role of Pre-medication Dental Evaluation in the prevention of MRONJ. Oral Surg Oral Med Oral Pathol Oral Radiol. 2018;125(5):440–5. 29580668 10.1016/j.oooo.2018.02.003 PMC7518027

[33] Decaux J, Magremanne M. Medication-related osteonecrosis of the jaw related to epacadostat and pembrolizumab. J Stomatol Oral Maxillofac Surg. 2020;121(6):740–2. 32413422 10.1016/j.jormas.2020.05.001

[34] Vallina C, Ramírez L, Torres J, Casañas E, Hernández G, López-Pintor RM. Osteonecrosis of the jaws produced by sunitinib: a systematic review. Med Oral Patol Oral Cir Bucal. 2019;24(3):e326–38. 31011143 10.4317/medoral.22858 PMC6530944

[35] Yamamoto D, Tsubota Y, Utsunomiya T, Sueoka N, Ueda A, Endo K, et al. Osteonecrosis of the jaw associated with everolimus: a case report. Mol Clin Oncol. 2017;6(2):255–7. 28357105 10.3892/mco.2016.1100 PMC5351763

[36] Granate-Marques A, Polis-Yanes C, Seminario-Amez M, Jané-Salas E, López-López J. Medication-related osteonecrosis of the jaw associated with implant and regenerative treatments: systematic review. Med Oral Patol Oral Cir Bucal. 2019;24(2):e195-203. 30818312 10.4317/medoral.22691 PMC6441601

[37] Bolette A, Lecloux G, Rompen E, Albert A, Kerckhofs G, Lambert F. Influence of induced infection in medication-related osteonecrosis of the jaw development after tooth extraction: a study in rats. J Craniomaxillofac Surg. 2019;47(2):349–56. 30595476 10.1016/j.jcms.2018.08.011

[38] Otto S, Tröltzsch M, Jambrovic V, Panya S, Probst F, Ristow O, et al. Tooth extraction in patients receiving oral or intravenous bisphosphonate administration: a trigger for BRONJ development? J Craniomaxillofac Surg. 2015;43(6):847–54. 25958767 10.1016/j.jcms.2015.03.039

[39] Cavalcante RC, Tomasetti G. Pentoxifylline and tocopherol protocol to treat medication-related osteonecrosis of the jaw: a systematic literature review. J Craniomaxillofac Surg. 2020;48(11):1080–6. 32998853 10.1016/j.jcms.2020.09.008

[40] Patel V, Gadiwalla Y, Sassoon I, Sproat C, Kwok J, McGurk M. Prophylactic use of pentoxifylline and tocopherol in patients who require dental extractions after radiotherapy for cancer of the head and neck. Br J Oral Maxillofac Surg. 2016;54(5):547–50. 26975577 10.1016/j.bjoms.2016.02.024

[41] Hadaya D, Soundia A, Freymiller E, Grogan T, Elashoff D, Tetradis S, et al. Nonsurgical management of medication-related osteonecrosis of the jaws using local wound care. J Oral Maxillofac Surg. 2018;76(11):2332–9. 29932939 10.1016/j.joms.2018.05.025 PMC6265090

[42] Katsarelis H, Shah NP, Dhariwal DK, Pazianas M. Infection and medication-related osteonecrosis of the jaw. J Dent Res. 2015;94(4):534–9. 25710950 10.1177/0022034515572021

[43] Soutome S, Otsuru M, Hayashida S, Murata M, Yanamoto S, Sawada S, et al. Relationship between tooth extraction and development of medication-related osteonecrosis of the jaw in cancer patients. Sci Rep. 2021;26(11):17226. 10.1038/s41598-021-96480-8 PMC8390686 34446755

[44] Otto S, Aljohani S, Fliefel R, Ecke S, Ristow O, Burian E, et al. Infection as an important factor in medication-related osteonecrosis of the jaw (MRONJ). Medicina (Kaunas). 2021;57(5):463. 34065104 10.3390/medicina57050463 PMC8151678

[45] Aghaloo TL, Cheong S, Bezouglaia O, Kostenuik P, Atti E, Dry SM, et al. RANKL inhibitors induce osteonecrosis of the jaw in mice with periapical disease. J Bone Miner Res. 2014;29(4):843–54. 24115073 10.1002/jbmr.2097 PMC4476544

[46] Marx RE. Drug-induced osteonecrosis of the jaws: how to diagnose, prevent, and treat it. Incorporated: Quintessence Publishing Company; 2021. p. 93.

[47] Sacco R, Woolley J, Yates J, Calasans-Maia MD, Akintola O, Patel V. The role of antiresorptive drugs and medication-related osteonecrosis of the jaw in nononcologic immunosuppressed patients: a systematic review. J Res Med Sci: Off J Isfahan Univ Med Sci. 2021; 26. https://www.ncbi.nlm.nih.gov/pmc/articles/PMC8240545/. 10.4103/jrms.JRMS_794_20 PMC8240545 34221052

[48] Kabilova TO, Kovtonyuk LV, Zonov EV, Ryabchikova EI, Popova NA, Nikolin VP, et al. Immunotherapy of hepatocellular carcinoma with small double-stranded RNA. BMC Cancer. 2014;14(1):338. 24886485 10.1186/1471-2407-14-338 PMC4038722

[49] McGowan K, McGowan T, Ivanovski S. Risk factors for medication-related osteonecrosis of the jaws: a systematic review. Oral Dis. 2018;24(4):527–36. 28656643 10.1111/odi.12708

[50] Varoni EM, Lombardi N, Villa G, Pispero A, Sardella A, Lodi G. Conservative management of Medication-Related Osteonecrosis of the Jaws (MRONJ): a retrospective cohort study. Antibiotics (Basel). 2021;10(2):195. 33671429 10.3390/antibiotics10020195 PMC7922963

[51] Mauceri R, Coniglio R, Abbinante A, Carcieri P, Tomassi D, Panzarella V, et al. The preventive care of medication-related osteonecrosis of the jaw (MRONJ): a position paper by Italian experts for dental hygienists. Support Care Cancer. 2022;30(8):6429–40. 35292850 10.1007/s00520-022-06940-8 PMC9213300

[52] Sim IW, Borromeo GL, Tsao C, Hardiman R, Hofman MS, Papatziamos Hjelle C, et al. Teriparatide promotes bone healing in medication-related osteonecrosis of the jaw: a placebo-controlled, randomized trial. J Clin Oncol. 2020;38(26):2971–80. 32614699 10.1200/JCO.19.02192

[53] Krege JH, Gilsenan AW, Komacko JL, Kellier-Steele N. Teriparatide and osteosarcoma risk: history, science, elimination of boxed warning, and other label updates. JBMR Plus. 2022;6(9):e10665. 36111201 10.1002/jbm4.10665 PMC9465003

[54] Shim GJ, Ohe JY, Yoon YJ, Kwon YD, Kim DY. Current trends in adjuvant therapies for medication-related osteonecrosis of the jaw. Appl Sci. 2022;12(8):4035. 10.3390/app12084035

[55] Morishita K, Yamada SI, Kawakita A, Hashidume M, Tachibana A, Takeuchi N, et al. Treatment outcomes of adjunctive teriparatide therapy for medication-related osteonecrosis of the jaw (MRONJ): a multicenter retrospective analysis in Japan. J Orthop Sci. 2020;25(6):1079–83. 32111549 10.1016/j.jos.2020.01.012

[56] Ferreira LDS, Abreu LG, Calderipe CB, Martins MD, Schuch LF, Vasconcelos ACU. Is teriparatide therapy effective for medication-related osteonecrosis of the jaw? a systematic review and meta-analysis. Oral Surg Oral Med Oral Pathol Oral Radiol. 2022;134(3):e229. 10.1016/j.oooo.2022.01.731 34331067

[57] RAPTOR: Randomised Controlled Trial of PENTOCLO in Mandibular Osteoradionecrosis - NIHR Funding and Awards. https://fundingawards.nihr.ac.uk/award/NIHR131050. 10.1186/s13063-025-08966-9 PMC12291413 40708048

[58] Heifetz-Li JJ, Abdelsamie S, Campbell CB, Roth S, Fielding AF, Mulligan JP. Systematic review of the use of pentoxifylline and tocopherol for the treatment of medication-related osteonecrosis of the jaw. Oral Surg Oral Med Oral Pathol Oral Radiol. 2019;128(5):491-497.e2. 31488389 10.1016/j.oooo.2019.08.004

[59] Russmueller G, Seemann R, Weiss K, Stadler V, Speiss M, Perisanidis C, et al. The association of medication-related osteonecrosis of the jaw with Actinomyces spp. infection. Sci Rep. 2016;6:31604. 27530150 10.1038/srep31604 PMC4987681

[60] Moretti F, Pelliccioni GA, Montebugnoli L, Marchetti C. A prospective clinical trial for assessing the efficacy of a minimally invasive protocol in patients with bisphosphonate-associated osteonecrosis of the jaws. Oral Surg Oral Med Oral Pathol Oral Radiol Endod. 2011;112(6):777–82. 22000426 10.1016/j.tripleo.2011.07.004

[61] Khan AA, Morrison A, Hanley DA, Felsenberg D, McCauley LK, O’Ryan F, et al. Diagnosis and management of osteonecrosis of the jaw: a systematic review and international consensus. J Bone Miner Res. 2015;30(1):3–23. 25414052 10.1002/jbmr.2405

[62] Freiberger JJ, Padilla-Burgos R, Chhoeu AH, Kraft KH, Boneta O, Moon RE, et al. Hyperbaric oxygen treatment and bisphosphonate-induced osteonecrosis of the jaw: a case series. J Oral Maxillofac Surg. 2007;65(7):1321–7. 17577496 10.1016/j.joms.2007.03.019

[63] Agrillo A, Fabio F, Ramieri V, Riccardi E, Quarato D, Rinna C, et al. Bisphosphonate-related osteonecrosis of the jaw (BRONJ): 5 year experience in the treatment of 131 cases with ozone therapy. Eur Rev Med Pharmacol Sci. 2012;1(16):1741–8. 23161050

[64] Min SH, Kang NE, Song SI, Lee JK. Regenerative effect of recombinant human bone morphogenetic protein-2/absorbable collagen sponge (rhBMP-2/ACS) after sequestrectomy of medication-related osteonecrosis of the jaw (MRONJ). J Korean Assoc Oral Maxillofac Surg. 2020;46(3):191–6. 32606280 10.5125/jkaoms.2020.46.3.191 PMC7338633

[65] Chen D, Zhao M, Mundy GR. Bone morphogenetic proteins. Growth Factors. 2004;22(4):233–41. 15621726 10.1080/08977190412331279890

[66] Robijns J, Nair RG, Lodewijckx J, Arany P, Barasch A, Bjordal JM, et al. Photobiomodulation therapy in management of cancer therapy-induced side effects: WALT position paper 2022. Front Oncol. 2022. 10.3389/fonc.2022.927685. 36110957 10.3389/fonc.2022.927685 PMC9468822

[67] Nica D, Rivis M, Roi C, Todea C, Duma VF, Sinescu C. Complementarity of photo-biomodulation, surgical treatment, and antibiotherapy for medication-related osteonecrosis of the jaws (MRONJ). Medicina (Kaunas). 2021;5(57):145. 10.3390/medicina57020145 PMC7914693 33562600

[68] Zheng Y, Dong X, Chen S, He Y, An J, Liu M, et al. Low-level laser therapy prevents medication-related osteonecrosis of the jaw-like lesions via IL-1RA-mediated primary gingival wound healing. BMC Oral Health. 2023;23(1):14. 36627695 10.1186/s12903-022-02678-1 PMC9832759

[69] Epstein JB, Arany PR, Yost SE, Yuan Y. Medication-related osteonecrosis of the jaw: successful medical management of complex maxillary alveolus with sinus involvement. Case Rep Oncol. 2023;16(1):412–28. 10.1159/000529502 PMC10294216 37384201

[70] Maines E, Monti E, Doro F, Morandi G, Cavarzere P, Antoniazzi F. Children and adolescents treated with neridronate for osteogenesis imperfecta show no evidence of any osteonecrosis of the jaw. J Bone Miner Metab. 2012;30(4):434–8. 22065238 10.1007/s00774-011-0331-3

[71] Wutzl A, Pohl S, Sulzbacher I, Seemann R, Lauer G, Ewers R, Drach J, Klug C. Factors influencing surgical treatment of bisphosphonate-related osteonecrosis of the jaws. Head Neck. 2012;34:194–200. 21400630 10.1002/hed.21708

[72] Schubert M, Klatte I, Linek W, Müller B, Döring K, Eckelt U, et al. The Saxon bisphosphonate register - therapy and prevention of bisphosphonate-related osteonecrosis of the jaws. Oral Oncol. 2012;48(4):349–54. 22130456 10.1016/j.oraloncology.2011.11.004

[73] Voss PJ, Joshi Oshero J, Kovalova-Müller A, Veigel Merino EA, Sauerbier S, Al-Jamali J, et al. Surgical treatment of bisphosphonate-associated osteonecrosis of the jaw: technical report and follow up of 21 patients. J Craniomaxillofac Surg. 2012;40(8):719–25. 22336489 10.1016/j.jcms.2012.01.005

[74] Pautke C, Bauer F, Otto S, Tischer T, Steiner T, Weitz J, et al. Fluorescence-guided bone resection in bisphosphonate-related osteonecrosis of the jaws: first clinical results of a prospective pilot study. J Oral Maxillofac Surg. 2010;1(69):84–91. 10.1016/j.joms.2010.07.014 20971542

[75] Marcianò A, Guzzo GM, Peditto M, Picone A, Oteri G. Medication-related osteonecrosis of the jaws and CDK4/6 inhibitors: a recent association. Int J Environ Res Public Health. 2020;17(24):9509. 33353034 10.3390/ijerph17249509 PMC7767118

[76] Sacco R, Sacco N, Hamid U, Ali SH, Singh M, Blythe JSJ. Microsurgical reconstruction of the jaws using vascularised free flap technique in patients with medication-related osteonecrosis: a systematic review. Biomed Res Int. 2018;2018:9858921. 29977926 10.1155/2018/9858921 PMC6011121

[77] Qaisi M, Montague L. Bone margin analysis for osteonecrosis and osteomyelitis of the jaws. Oral Maxillofac Surg Clin North Am. 2017;29(3):301–13. 28709531 10.1016/j.coms.2017.03.007

[78] Lopes RN, Rabelo GD, Rocha AC, Carvalho PAG, Alves FA. Surgical therapy for bisphosphonate-related osteonecrosis of the jaw: six-year experience of a single institution. J Oral Maxillofac Surg. 2015;73(7):1288–95. 25871903 10.1016/j.joms.2015.01.008

[79] Raber-Durlacher JE, Elad S, Barasch A. Oral mucositis. Oral Oncol. 2010;46(6):452–6. 20403721 10.1016/j.oraloncology.2010.03.012

[80] Migliorati CA, Epstein JB, Abt E, Berenson JR. Osteonecrosis of the jaw and bisphosphonates in cancer: a narrative review. Nat Rev Endocrinol. 2011;7(1):34–42. 21079615 10.1038/nrendo.2010.195

[81] Migliorati CA, Casiglia J, Epstein J, Jacobsen PL, Siegel MA, Woo SB. Managing the care of patients with bisphosphonate-associated osteonecrosis: an American Academy of Oral Medicine position paper. J Am Dent Assoc. 2005;136(12):1658–68. 16383047 10.14219/jada.archive.2005.0108

[82] Ripamonti CI, Maniezzo M, Campa T, Fagnoni E, Brunelli C, Saibene G, et al. Decreased occurrence of osteonecrosis of the jaw after implementation of dental preventive measures in solid tumour patients with bone metastases treated with bisphosphonates. The experience of the National Cancer Institute of Milan. Ann Oncol. 2009;20(1):137–45. 18647964 10.1093/annonc/mdn526

[83] Campisi G, Russo L, Agrillo A, Vescovi P, Fusco V, Bedogni A. BRONJ expert panel recommendation of the Italian Societies for Maxillofacial Surgery (SICMF) and Oral Pathology and Medicine (SIPMO) on Bisphosphonate-Related Osteonecrosis of the Jaws: risk assessment, preventive strategies and dental management. It J Maxillofac Surg. 2011;1(22):103–24.

[84] Vandone AM, Donadio M, Mozzati M, Ardine M, Polimeni MA, Beatrice S, et al. Impact of dental care in the prevention of bisphosphonate-associated osteonecrosis of the jaw: a single-center clinical experience. Ann Oncol. 2012;23(1):193–200. 21427065 10.1093/annonc/mdr039

[85] Beth-Tasdogan NH, Mayer B, Hussein H, Zolk O, Peter JU. Interventions for managing medication-related osteonecrosis of the jaw. Cochrane Database Syst Rev. 2022;7(7):CD012432. 35866376 10.1002/14651858.CD012432.pub3 PMC9309005

[86] Bedogni A, Mauceri R, Fusco V, Bertoldo F, Bettini G, Fede OD, et al. Italian Position Paper (SIPMO-SICMF) on Medication-Related Osteonecrosis of the Jaw (MRONJ). Qeios. 2023 Mar 27. https://www.qeios.com/read/PBUJ6Z. 10.1111/odi.14887 38317291

[87] Beth-Tasdogan NH, Mayer B, Hussein H, Zolk O, Peter JU. Interventions for managing medication‐related osteonecrosis of the jaw. Cochrane Database Syst Rev. 2022; 2022(7). https://www.readcube.com/articles/10.1002%2F14651858.cd012432.pub3. 10.1002/14651858.CD012432.pub3 PMC9309005 35866376

[88] Yarom N, Shapiro CL, Peterson DE, Van Poznak CH, Bohlke K, Ruggiero SL, et al. Medication-related osteonecrosis of the jaw: MASCC/ISOO/ASCO clinical practice guideline. JCO. 2019;37(25):2270–90. 10.1200/JCO.19.01186 31329513

[89] Cabras M, Gambino A, Broccoletti R, Sciascia S, Arduino PG. Lack of evidence in reducing risk of MRONJ after teeth extractions with systemic antibiotics. J Oral Sci. 2021;63(3):217–26. 34193777 10.2334/josnusd.21-0016

[90] Campisi G, Mauceri R, Bertoldo F, Bettini G, Biasotto M, Colella G, et al. Medication-Related Osteonecrosis of Jaws (MRONJ) prevention and diagnosis: Italian consensus update 2020. Int J Environ Res Public Health. 2020;17(16):5998. 32824826 10.3390/ijerph17165998 PMC7460511

[91] Bramati A, Girelli S, Farina G, Dazzani MC, Torri V, Moretti A, et al. Prospective, mono-institutional study of the impact of a systematic prevention program on incidence and outcome of osteonecrosis of the jaw in patients treated with bisphosphonates for bone metastases. J Bone Miner Metab. 2015;33(1):119–24. 24553860 10.1007/s00774-014-0566-x

[92] Trosman JR, Carlos RC, Simon MA, Madden DL, Gradishar WJ, Benson AB, et al. Care for a patient with cancer as a project: management of complex task interdependence in cancer care delivery. J Oncol Pract. 2016;12(11):1101–13. 27577619 10.1200/JOP.2016.013573 PMC5455414

[93] Bledsaw K, Prudowsky ZD, Yang E, Harriehausen CX, Robins J, DeJean J, et al. A novel oncodental collaborative team: integrating expertise for central line-associated bloodstream infection prevention in pediatric oncology patients. JCO Oncol Pract. 2023;19(1):e25-32. 36137251 10.1200/OP.22.00302

[94] Mücke T, Deppe H, Hein J, Wolff KD, Mitchell DA, Kesting MR, et al. Prevention of bisphosphonate-related osteonecrosis of the jaws in patients with prostate cancer treated with zoledronic acid – a prospective study over 6 years. J Cranio-Maxillofac Surg. 2016;44(10):1689–93. 10.1016/j.jcms.2016.07.026 27555374

[95] Dimopoulos MA, Kastritis E, Bamia C, Melakopoulos I, Gika D, Roussou M, et al. Reduction of osteonecrosis of the jaw (ONJ) after implementation of preventive measures in patients with multiple myeloma treated with zoledronic acid. Ann Oncol. 2009;20(1):117–20. 18689864 10.1093/annonc/mdn554

[96] Montefusco V, Gay F, Spina F, Miceli R, Maniezzo M, Teresa Ambrosini M, et al. Antibiotic prophylaxis before dental procedures may reduce the incidence of osteonecrosis of the jaw in patients with multiple myeloma treated with bisphosphonates. Leuk Lymphoma. 2008;49(11):2156–62. 19021059 10.1080/10428190802483778

[97] Kwon YD, Kim DY, Ohe JY, Yoo JY, Walter C. Correlation between serum C-terminal cross-linking telopeptide of type I collagen and staging of oral bisphosphonate-related osteonecrosis of the jaws. J Oral Maxillofac Surg. 2009;67(12):2644–8. 19925985 10.1016/j.joms.2009.04.067

[98] Ottesen C, Schiodt M, Jensen SS, Kofod T, Gotfredsen K. Tooth extractions in patients with cancer receiving high-dose antiresorptive medication: a randomized clinical feasibility trial of drug holiday versus drug continuation. Oral Surg Oral Med Oral Pathol Oral Radiol. 2022;133(2):165–73. 34275774 10.1016/j.oooo.2021.06.003

[99] Hoefert S, Yuan A, Munz A, Grimm M, Elayouti A, Reinert S. Clinical course and therapeutic outcomes of operatively and non-operatively managed patients with denosumab-related osteonecrosis of the jaw (DRONJ). J Craniomaxillofac Surg. 2017;45(4):570–8. 28238559 10.1016/j.jcms.2017.01.013

[100] Watters AL, Hansen HJ, Williams T, Chou JF, Riedel E, Halpern J, et al. Intravenous bisphosphonate-related osteonecrosis of the jaw: long-term follow-up of 109 patients. Oral Surg Oral Med Oral Pathol Oral Radiol. 2013;115(2):192–200. 23036797 10.1016/j.oooo.2012.05.017

[101] Liu C, Wang L, Liu L, Zhuang J, Tang S, Zhang T, et al. Efficacy and safety of de-escalation bone- modifying agents for cancer patients with bone metastases: a systematic review and meta-analysis. Cancer Manag Res. 2018;21(10):3809–23. 10.2147/CMAR.S176811 PMC6159799 30288112

[102] ESMO. De-escalation of commonly used bone-treating agents is a reasonable treatment option for patients with bone metastases from breast cancer. https://www.esmo.org/oncology-news/de-escalation-of-commonly-used-bone-treating-agents-is-a-reasonable-treatment-option-for-patients-with-bone-metastases-from-breast-cancer.

[103] Yao S, Ding X, Rong G, Zhou J, Zhang B. Association between malignant diseases and Medication-Related Osteonecrosis of the Jaw (MRONJ): a systematic review and meta-analysis. J Craniofac Surg. 2023;34(2):669-73. 36184756 10.1097/SCS.0000000000009033

[104] Gross J, Méder ZZ, De Dreu CKW, Romano A, Molenmaker WE, Hoenig LC. The evolution of universal cooperation. Sci Adv. 2023;9(7):eadd8289. 36800427 10.1126/sciadv.add8289 PMC9937576

[105] Zajac S, Woods A, Tannenbaum S, Salas E, Holladay CL. Overcoming challenges to teamwork in healthcare: a team effectiveness framework and evidence-based guidance. Front Commun. 2021. 10.3389/fcomm.2021.606445. 10.3389/fcomm.2021.606445

[106] Weller J, Boyd M, Cumin D. Teams, tribes and patient safety: overcoming barriers to effective teamwork in healthcare. Postgrad Med J. 2014;90(1061):149–54. 24398594 10.1136/postgradmedj-2012-131168

[107] Emanuele J, Koetter L. Workflow Opportunities and Challenges in Healthcare. 2007 BPM & Workflow Handbook. 2007;1(1):157.

[108] Harper RP, Fung E. Resolution of bisphosphonate-associated osteonecrosis of the mandible: possible application for intermittent low-dose parathyroid hormone [rhPTH(1–34)]. J Oral Maxillofac Surg. 2007;65(3):573–80. 17307613 10.1016/j.joms.2006.10.076

[109] Drudge-Coates L, Van den Wyngaert T, Schiødt M, van Muilekom HAM, Demonty G, Otto S. Preventing, identifying, and managing medication-related osteonecrosis of the jaw: a practical guide for nurses and other allied healthcare professionals. Support Care Cancer. 2020;28(9):4019–29. 32307659 10.1007/s00520-020-05440-x PMC7378104

